# Supplementary figures and images for: Comparative Transcriptomic and Metabolic Analyses Reveal the Coordinated Mechanisms in Pinus koraiensis under Different Light Stress Conditions
Source: Int J Mol Sci. 2022 Aug 23;23(17):9556. doi: 10.3390/ijms23179556 (PMC9455776; doi:10.3390/ijms23179556)

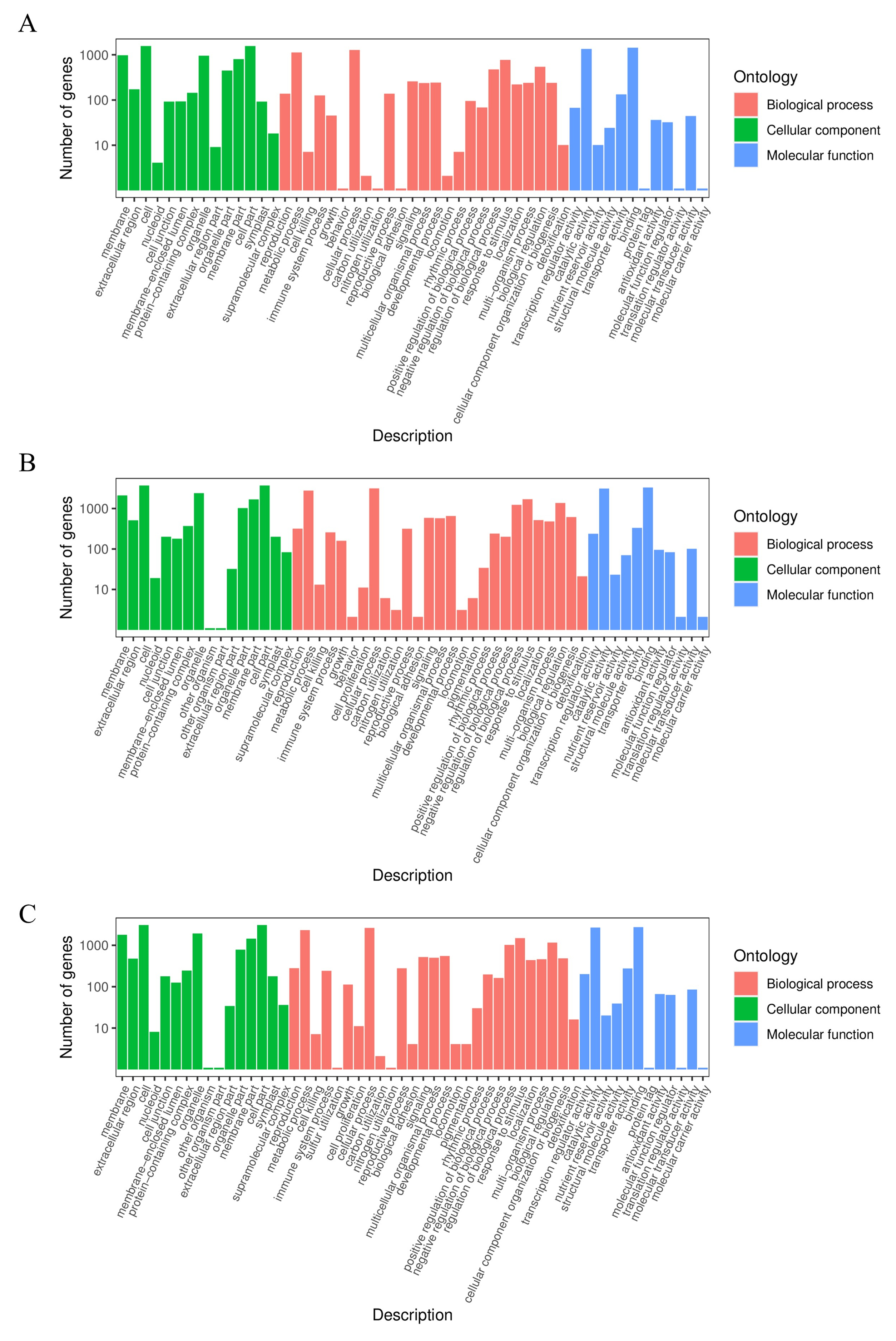

Supplement: Supplementary file 1 [file ijms-23-09556-s001.zip › Supplementary Figure/Supplementary Figure S1.jpg]

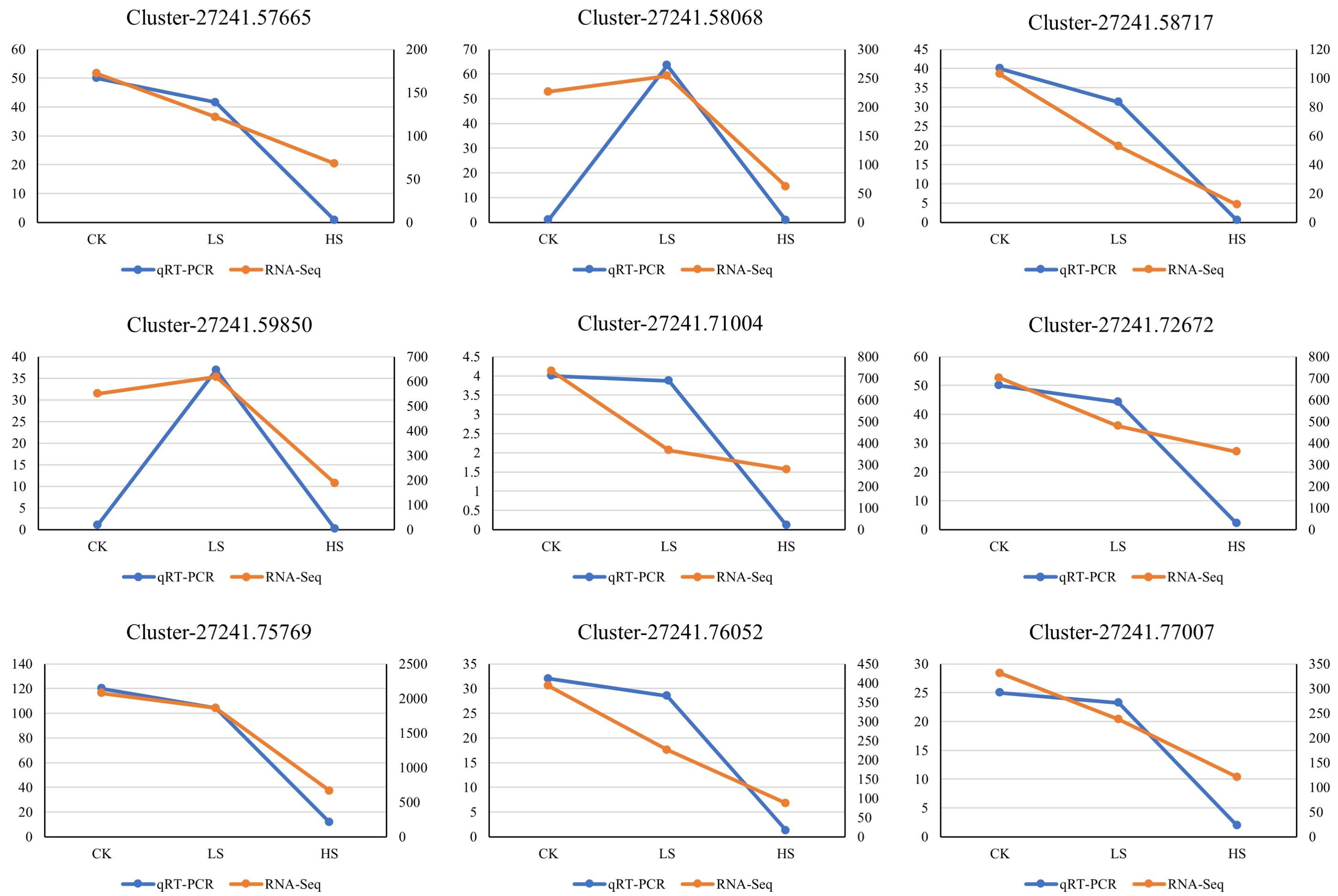

Supplement: Supplementary file 1 [file ijms-23-09556-s001.zip › Supplementary Figure/Supplementary Figure S2.jpg]
